# Research on electromagnetic compatibility analysis of automation equipment based on generative adversarial networks and pulse sparse convolution

**DOI:** 10.1371/journal.pone.0341052

**Published:** 2026-03-10

**Authors:** Wenrui Ding, Deren Feng

**Affiliations:** 1 School of Electronic Engineering and Optoelectronic Technology, Nanjing University of Science and Technology, Nanjing, Jiangsu, China; 2 China Electronic Product Reliability and Environmental Testing Institute, Guangzhou, Guangdong, China; National Institute of Technology, India (Institute of National Importance), INDIA

## Abstract

Electromagnetic interference (EMI) analysis in high-speed industrial systems is increasingly challenged by multi-gigahertz sampling rates, complex transient behaviors, and stringent real-time constraints. To address these challenges, this paper proposes a pulse-aware generative and analysis framework based on a generative adversarial network (GAN) combined with pulse sparse convolution using leaky integrate-and-fire (LIF) spiking neurons. A multi-scale discriminator and gradient penalty stabilization are employed to improve waveform generation fidelity, achieving a Fréchet distance (FID) of 0.72 and a global difference metric (GDM) of 0.18 ± 0.03 on an industrial-grade Electromagnetic compatibility (EMC) dataset. The proposed framework is further applied to crosstalk prediction, where it reduces pulse-width and phase prediction errors by more than 40% compared with classical numerical solvers such as finite-difference time-domain (FDTD), finite element method (FEM), and method of moments (MoM), and consistently outperforms representative learning-based EMC models. To enable real-time deployment, the pulse sparse convolution architecture is implemented on an field-programmable gate array (FPGA) platform using fixed-point arithmetic, achieving deterministic inference at 5 GS/s with a measured power consumption of 0.71 W. Extensive experiments on traction systems, industrial robots, CNC drives, photovoltaic inverters, and UAV (Unmanned Aerial Vehicle) electronics demonstrate that the proposed approach provides accurate, stable, and energy-efficient EMI analysis suitable for practical industrial EMC applications.

## 1. Introduction

The widespread application of automation equipment in modern industrial systems is facing increasingly severe EMC challenges. According to statistics, the signal bit error rate caused by EMI in high-speed train traction systems can reach up to 10−3, and industrial robots experience downtime due to cable crosstalk exceeding 120 hours annually. The economic losses caused by Electromagnetic compatibility (EMC) issues in smart manufacturing production lines amount to billions of yuan each year [1–3]. In complex electromagnetic environments, broadband noise (0.5-2.5 GHz) generated by high-frequency switching devices inside equipment propagates through multiple paths such as cable coupling and spatial radiation, leading to malfunctions in control systems, communication interruptions, and other accidents. Although traditional electromagnetic shielding and filtering solutions can mitigate some interference, their protective effectiveness significantly decreases in the face of higher frequency band interference (up to 6 GHz) brought by new technologies such as 5G communication and silicon carbide power devices [4–6]. More critically, existing international standards such as International Electrotechnical Commission (IEC) 61000−4 for testing limits of transient burst interference are no longer able to cover extreme scenarios in actual working conditions [7].

Current EMC research primarily relies on full-wave simulation and experimental verification, facing significant bottlenecks. The finite element method (FEM) for solving multi-conductor transmission line equations consumes hours to days and lacks adaptability to changes in wire harness topology [8,9]. Although experimental measurements can obtain real data, they are limited by the cost of sensor deployment and the difficulty of reproducing operating conditions, resulting in a high missing rate of key scenario samples, up to 65% [10]. It is worth noting that existing methods for characterizing interference mechanisms mostly focus on frequency domain energy distribution, failing to effectively explore transient features and correlation patterns in time domain waveforms. For example, the microsecond-level pulse waveform of pantograph-catenary offline disturbance contains rich coupling mode information, but traditional Fourier transform struggles to capture its non-stationary characteristics [11,12]. Furthermore, the optimization of cable layout inside equipment relies on engineers’ experience, lacking quantitative decision-making basis, leading to a protection scheme design cycle lasting several months [13].

Recent progress in spiking sequence modeling and event-driven neuromorphic acceleration offers complementary technical perspectives for low-power transient signal processing. SNN-BERT [14] introduces a spiking formulation for BERT-like sequence modeling by proposing Bidirectional Parallel Spiking Neurons (BPSN) together with an “individual coding” strategy that aligns the number of timesteps with the number of tokens, enabling parallel feature extraction across token positions and improving training efficiency in NLP settings. MENAGE [15] focuses on hardware-side event-driven efficiency and presents a CMOS-compatible mixed-signal neuromorphic accelerator: synaptic operations are implemented using an analog C2C ladder structure and neuron functions are realized with op-amp-based circuits; to improve utilization under event sparsity, MENAGE further introduces a “virtual neuron” execution concept (one neuron engine emulates multiple model neurons), complemented by memory-based event control and ILP-based mapping, and reports energy-efficiency results at a stated operating point (including up to 12.1 TOPS/W on a CIFAR10-DVS setting). A direct quantitative comparison with these works is not methodologically sound under the current EMC crosstalk prediction setting, because the problem formulations, input semantics, training objectives, and evaluation protocols differ (NLP token sequences and mixed-signal accelerator benchmarking, respectively). The cited baselines are therefore used to delineate methodological differences and to motivate potential extensions toward more general event coding and accelerator-mapping strategies for transient EMC waveform processing.

**Fig 1:**
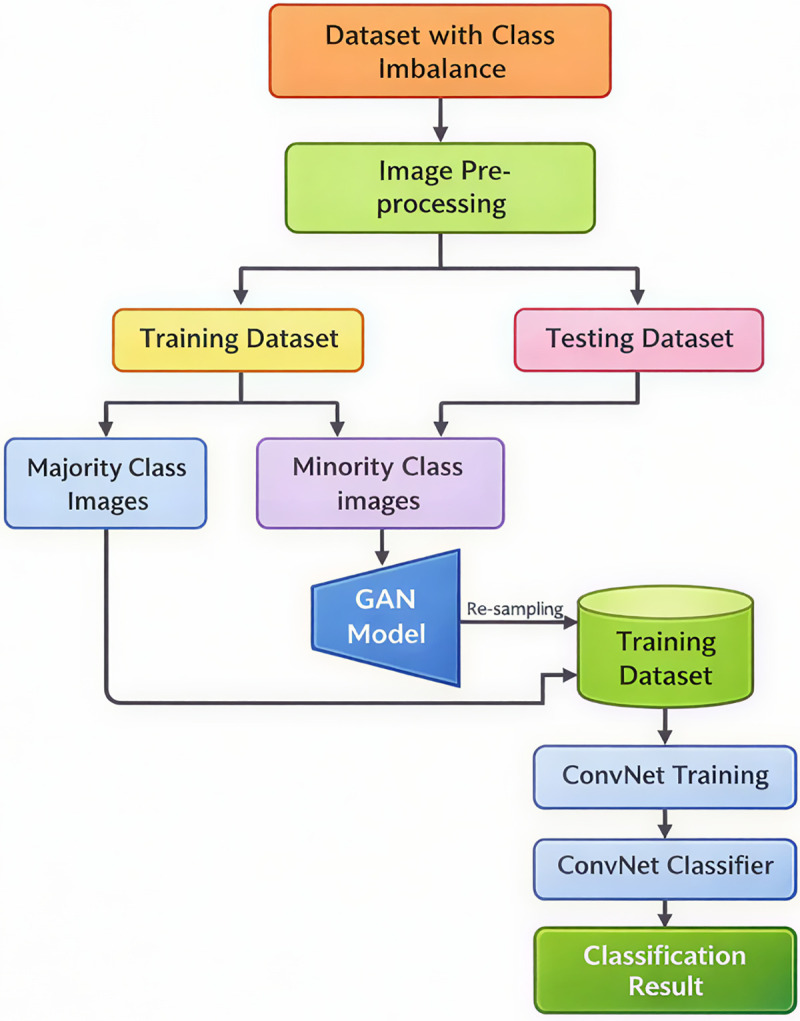
Flowchart of the overall framework.

Addressing the aforementioned challenges, this study proposes an innovative framework that integrates GANs with impulsive sparse convolution. By constructing a deep generative model of electromagnetic interference waveforms, it breaks through the bottleneck of scarce measured data. Leveraging the sparse event-driven characteristics of impulsive neural networks, it achieves millisecond-level real-time extraction of interference features. Combined with cross-scale electromagnetic coupling modeling, a dynamic optimization mechanism for cable layout inside equipment is established [16–19]. This framework deeply couples data-driven methods with physical mechanism models for the first time, providing quantifiable theoretical tools and engineering solutions for electromagnetic compatibility design of automated equipment. Fig 1 shows the key structure of the article.

To better position this study within recent EMC research, Table 1 summarizes representative prior works in related directions, including learning-based EMC/EMI recognition and prediction, physics-informed neural modeling for EMI reconstruction, generative modeling of electromagnetic signals, and event-driven/spiking implementations for low-power deployment. For each work, we highlight its main idea together with practical advantages and limitations, which motivates the proposed pulse-aware, physics-guided, hardware-deployable framework.

**Table 1:** Summary of representative prior works in EMC/EMI analysis.

Category	Representative work	Core technique	Main advantages	Main limitations
Classical full-wave solvers	Taflove & Hagness (2005) [20]	Finite-Difference Time-Domain (FDTD)	High physical accuracy; rigorous Maxwell-based modeling	Extremely high computational cost; unsuitable for real-time or large-scale industrial deployment
Learning-based EMC/EMI analysis	LeCun, Bengio, & Hinton (2015) [21]	Deep neural networks (DNNs)	Strong capability for data-driven feature learning	Lacks explicit physical constraints; generalization depends on data coverage
Physics-informed modeling	Raissi, Perdikaris, & Karniadakis (2019) [22]	Physics-Informed Neural Networks (PINNs)	Embeds governing equations; improved interpretability	High training complexity; limited scalability to high-frequency transient signals
Generative modeling of signals	Goodfellow et al. (2014) [23]	Generative Adversarial Networks (GANs)	Powerful generative capability for complex signal distributions	Physical consistency not guaranteed without constraints
Event-driven/ spiking computation	Maass (1997) [24]	Spiking neural networks (SNNs)	Event-driven processing; energy efficiency	Primarily explored for classification; limited application to waveform synthesis

Although generative adversarial networks (GANs) combined with spiking neural models have been explored in recent studies, most existing spiking-GAN hybrids are designed for image synthesis, neuromorphic vision, or probabilistic sampling. In these works, spiking neurons mainly serve as energy-efficient substitutes for conventional activations, and the learning objective is typically to approximate static data distributions in the spatial domain, with limited consideration of underlying physical mechanisms.In contrast, the proposed framework targets electromagnetic compatibility (EMC) analysis of automated equipment, focusing on microsecond-scale, highly non-stationary transient electromagnetic interference (EMI) waveforms. The novelty does not lie in a generic combination of GANs and spiking neurons, but in a task-oriented, physics-constrained redesign of both the generator and the discriminator. The main contributions are summarized as follows:

(1) Physically constrained, EMI-oriented GAN architecture: Multi-scale discriminators and waveform-level constraints are introduced to explicitly embed electromagnetic coupling characteristics, enabling accurate reconstruction of transient EMI pulses that are not covered by existing standards.
(2) Resonant neuron-based pulse modulation generator: A pulse modulation mechanism based on resonant leaky integrate-and-fire neurons is proposed to reproduce damped oscillations and resonance behaviors observed in real EMI phenomena, aligning neuronal dynamics with physical resonance frequencies.
(3) Pulse-sparse convolution for EMC feature extraction rather than generation: Unlike prior spiking-GAN hybrids that confine spiking neurons to the generative process, this work employs pulse-sparse convolution as an independent feature extraction and analysis module, supporting real-time EMC characterization and ultra-low-power deployment.
(4) Closed-loop coupling with EMC-driven engineering optimization: The generated pulses and extracted features are further integrated into a closed-form cable crosstalk model and routing optimization, establishing a closed loop from data generation and perception to engineering decision-making.

Through these distinctions, this study extends spiking-GAN concepts from generic data modeling to a physically interpretable, engineering-oriented EMC analysis framework, representing a substantive methodological departure from existing hybrid approaches.

The remainder of this paper is organized as follows: Section 2 presents the modeling of typical electromagnetic interference scenarios and describes the construction of an industrial-grade EMC dataset. Section 3 details the proposed pulse-aware generative and analysis framework, including the multi-scale GAN architecture, pulse modulation generator, and pulse sparse convolution–based feature extraction. Section 4 focuses on the hardware implementation and real-time deployment of the proposed method on an FPGA platform, covering architectural design, resource mapping, and power-performance evaluation. Section 5 introduces the crosstalk-constrained routing optimization model and its integration with electromagnetic compatibility theory. Section 6 provides comprehensive experimental validation and performance comparisons across multiple industrial application scenarios. Finally, Section 7 concludes the paper and outlines directions for future research.

## 2. Interference modeling and coupling mechanism

### 2.1. Modeling of typical interference scenarios

**Fig 2:**
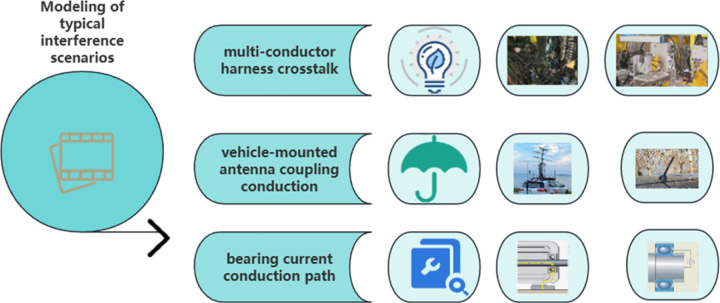
Typical interference scenario.


As shown in Fig 2, the electromagnetic compatibility issues of automation equipment primarily stem from three typical interference scenarios: multi-conductor harness crosstalk, vehicle-mounted antenna coupling conduction, and bearing current conduction path [25]. The multi-conductor transmission line equation precisely describes the electromagnetic energy coupling mechanism between cables, and its frequency domain expression is as follows:

$$\frac{\partial }{\partial_{z}}\left[\begin{array}{c} {}*20cV\left(z,\omega \right)\\ I\left(z,\omega \right) \end{array}\right]=\left[\begin{array}{cc} {}*20c0 & -Z\left(\omega \right)\\ -Y\left(\omega \right) & 0 \end{array}\right]\left[\begin{array}{c} {}*20cV\left(z,\omega \right)\\ I\left(z,\omega \right) \end{array}\right]$$ (1)

The impedance matrix Z(ω)=R+jωL and admittance matrix Y(ω)=G+jωC respectively characterize the resistance and inductance effects of conductors, as well as the conductivity and capacitance characteristics of dielectrics. The mutual capacitance of dense wiring harnesses inside industrial robot control cabinets $C_{m}$ can reach up to 50pF/m, and the mutual inductance $L_{m}$ is approximately 0.3 μH/m, leading to significantly enhanced crosstalk in frequency bands above 30MHz [26].

The offline interference from pantograph-catenary system threatens the on-board communication system through a radiation-conduction coupling path, and its electromagnetic coupling coefficient can be modeled as follows:


$$\begin{array}{c} K_{c}\left(f\right)=\frac{1}{T}\int_{0}^{T}\left|\iint {\mathrm{\backslash nolimits}}_{\Sigma }E_{rad}\left(r,t\right)\times H_{ant}^{*}\left(r,f\right)dS\right|dt \end{array}$$ (2)

This formula quantifies the spatial integral correlation between the time-varying radiation field $E_{rad}$ and the antenna pattern function $H_{ant}$. The transient electric field intensity generated by the pantograph of a high-speed train when it is offline exceeds 120dBμV/m at the 200MHz frequency point, which couples through the GSM-R antenna port to form in-band interference as high as -35dBm, leading to a sharp increase in communication bit error rate [27].

The bearing current conduction path forms a closed interference loop, and its conducted disturbance voltage satisfies a differential equation:


$$V_{bearing}\left(t\right)=\sum {\mathrm{\backslash nolimits}}_{k=1}^{N}\alpha_{k}e^{-\beta_{k}\left(t-\tau_{k}\right)}\cos\left(2\pi f_{k}\left(t-\tau_{k}\right)+\varphi_{k}\right)\cdot u\left(t-\tau_{k}\right)$$ (3)

This model characterizes the conduction characteristics of pulse trains generated by the switching devices of the induction system through the mechanical structure. $f_{k}\in \left[1,100\right]MHz$ Actual measurements show that when a frequency converter drives a motor, the peak longitudinal voltage across the bearings exceeds 100V. Pulses with a pulse width of 2μs form mA-level discharge currents through parasitic capacitance, accelerating the electrical erosion damage to the bearings [28].

### 2.2. Construction of industrial-grade EMC dataset

To ensure sufficient data diversity, the industrial-grade EMC dataset was constructed across multiple representative application scenarios, including high-speed rail traction converters, industrial robot control cabinets, photovoltaic inverters, and motor drive systems. For each scenario, measurements were performed under varied operating conditions, such as different load levels, switching frequencies, modulation strategies, and cable configurations. This design enables the dataset to cover a broad range of conducted and radiated interference characteristics, spanning both low-frequency harmonics and high-frequency transient pulses up to the gigahertz range.

All measurements were conducted on standardized EMC test platforms compliant with IEC 62236-3. Calibrated high-bandwidth current probes and near-field probes were deployed at predefined ports, and waveform acquisition was performed using oscilloscopes with sampling rates up to 5 GS/s and a dynamic range exceeding 80 dB. Environmental parameters, including temperature and supply voltage, were kept within controlled ranges to ensure recording consistency and repeatability across experiments.

To guarantee ground truth integrity, interference sources were isolated using a controlled excitation and sequential activation protocol. During data acquisition, only one dominant noise source—such as inverter switching, bearing discharge, or cable crosstalk—was intentionally excited at a time, while other potential interference paths were physically disconnected or electrically suppressed. As a result, waveform labeling was based on known excitation conditions, circuit topology, and synchronized trigger signals, rather than post hoc statistical inference. This physics-guided acquisition and labeling strategy ensures a clear one-to-one correspondence between recorded waveforms and their originating noise sources, providing reliable ground truth for GAN training and subsequent pulse-based feature extraction.

To ensure physical consistency, the dataset construction was guided by established electromagnetic coupling theory and practical EMC measurement experience. Data acquisition was designed in consultation with EMC engineers to isolate dominant interference paths and to enforce known physical constraints, such as bounded pulse widths, monotonic spectral energy decay, and impedance-consistent coupling behavior. As a result, the recorded waveforms inherently reflect Maxwell-compatible propagation characteristics, providing a physically meaningful foundation for GAN training.

A high-frequency current probe and a 5GS/s sampling rate oscilloscope system are deployed at the DC bus port to capture the broadband noise generated during the switching on and off process of switching devices:


$$i_{noise}\left(t\right)=\sum {\mathrm{\backslash nolimits}}_{k=1}^{N}A_{k}e^{-\frac{t-\tau_{k}}{\sigma k}}\cos\left(2\pi fk\left(t-\tau_{k}\right)+\varphi_{k}\right)\cdot u\left(t-\tau_{k}\right)$$ (4)

This model characterizes the damped oscillation waveform excited by the switching events of insulated-gate bipolar transistor (IGBT) modules, which is $A_{k}$ positively correlated with the rate of change of switching current and $f_{k}$ determined by parasitic parameter resonance. A total of 1500 sets of effective data have been collected, with a dynamic range of 80dB, covering the frequency band from 0 to 2.5GHz [29].

The generation of adversarial networks for synthesizing bearing fault current waveforms addresses the issue of sample scarcity. The generator network maps Gaussian noise to time-domain waveforms through five layers of transposed convolution, while the discriminator employs a multi-scale feature extraction structure:


$$L_{adv}=E_{x\sim p_{data}}\left[\log D\left(x\right)\right]+E_{z\sim p_{z}}\left[\log\left(1-D\left(G\left(z\right)\right)\right)\right]+\lambda E_{x\sim p_{z}}\left[{\left\|\nabla_{x}D\left(x\right)\right\|}_{2}^{2}\right]$$ (5)

The gradient penalty term λ is set to 0.3 to stabilize the training process. The generated samples are verified through feature selection, and the global discrepancy with the measured data is less than 0.25, with the pulse width error controlled within 0.1μs [30–32].

The raw signal from the vibration sensor needs to be converted into a sparse pulse sequence to accommodate the neuromorphic computing architecture. Based on the integration-and-firing mechanism, the timing of the trigger pulse for the vibration envelope signal v(t) is determined by threshold conditions:


$$s\left(t\right)=\Sigma_{t_{i}}\delta \left(t-t_{i}\right),t_{i}=\inf\left\{t|\int_{0}^{t}v\left(\tau \right)d\tau \geq V_{th}\right\}$$ (6)

During $V_{th}=0.2V\cdot s$ setting, the pulse emission rate after encoding the typical bearing vibration signal is approximately 12kHz, with an information compression rate of 98%, effectively retaining the 2-8kHz fault feature frequency band.

### 2.3. Permits and approvals

The study reported in this manuscript did not involve field sampling, access to protected or restricted sites, human participants, or animal subjects. All data were obtained through laboratory-based measurements, numerical simulations, and computational analyses conducted within institutional research facilities. Therefore, no permits or approvals from external authorities were required for this work.

## 3. Cross-scale generation recognition architecture

### 3.1. Multi-scale discriminator structure optimization

Traditional GANs face the issue of a single receptive field in discriminators when modeling EMC data, making it difficult to simultaneously capture both the macroscopic envelope characteristics and microscopic transient details of interference waveforms. To address this, a multi-scale feature fusion architecture is proposed, deploying parallel convolutional paths at the input end:


$$F_{k}=\sigma \left(W_{k}^{conv}*X+b_{k}\right),k\in \left\{1,2,3\right\}$$ (7)

The corresponding time resolution ranges from 0.5μs to 5μs [33]. The outputs from each path are compressed using depthwise separable convolution and then concatenated to form a mixed feature tensor:


$$H=Concat\left[DepthwiseConv\left(F_{1}\right),Point\;wiseConv\left(F_{2}\right),DepthwiseConv\left(F_{3}\right)\right]$$ (8)

This structure enables the discriminator to identify both the periodic characteristics of millisecond-level pulse trains and the nanosecond-level rising edge oscillation details when analyzing pantograph-catenary disturbance data [34].

Addressing the multi-task conflict in authenticity judgment and interference type classification within electromagnetic compatibility data, a task decoupling mechanism is designed. Feature splitting based on width calculation is implemented at the feature level:


$$h_{real}=\varphi \left(\sum {\mathrm{\backslash nolimits}}_{i=1}^{C}w_{i}^{real}\cdot H_{:,i}\right),h_{class}=\varphi \left(\sum {\mathrm{\backslash nolimits}}_{j=1}^{C}w_{j}^{class}\cdot H_{:,j}\right)$$ (9)

The weight vector w is adaptively generated through a gated unit, and the balance coefficient α=0.7 controls the task correlation strength. This structure enables the discriminator to achieve an accuracy of 92.3% in the classification branch during training, while reducing the Fréchet distance of generated data to 14.7 [35–37].

The gradient penalty strategy is introduced to stabilize the training process of the multi-scale discriminator. A Lipschitz constraint is imposed on the real data manifold:


$$L_{GP}=\lambda E_{\hat{x}\sim P_{x}}\left[{\left({\left\|\nabla_{\hat{x}}D\left(\hat{x}\right)\right\|}_{2}-1\right)}^{2}\right]$$ (10)

Among them $\hat{x}=\in x_{real}+\left(1-\in \right)x_{fake}$, interpolation samples and penalty factors λ=0.3. This constraint reduces the occurrence rate of mode collapse in the training of bearing current data to below 5% [38–40].

### 3.2. Design of pulse modulation generator

**Fig 3:**
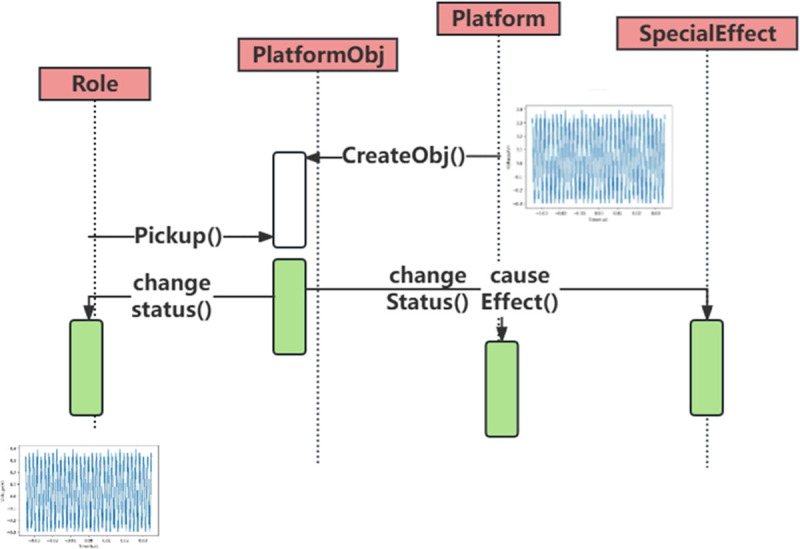
Pulse modulation generator design.

As shown in Fig 3, the traditional generators struggle to accurately reproduce the non-stationary transient characteristics of electromagnetic interference waveforms. Although the proposed generator is data-driven, its design is constrained by physically observed EMI behaviors. The resonant neuron model is parameterized to match measured damping factors and resonance frequency ranges, preventing the generation of non-physical oscillations. During validation, generated waveforms are screened using physics-derived indicators, including pulse width bounds, spectral roll-off characteristics, and coupling trends consistent with transmission-line and radiation models. This ensures that statistically realistic samples also remain physically plausible.

To address this, a pulse modulation architecture based on resonant neurons is proposed. The core of the generator employs a leaky integrate-and-fire-resonant neuron model:


$$\tau_{m}\frac{dV_{j}}{dt}=-V_{j}+\beta \frac{d^{2}V_{j}}{dt^{2}}+\sum {\mathrm{\backslash nolimits}}_{i}w_{ij}\sum {\mathrm{\backslash nolimits}}_{t_{i}}e^{-\frac{t-t_{j}}{\tau_{s}}}$$ (11)

The time constant $\tau_{m}=5ms$ controls the decay rate of membrane potential, the resonance factor β=0.4 enhances the response ability in specific frequency bands, and presynaptic pulses are input after being modulated by an exponential kernel $\tau_{s}=2ms$. When generating bearing current data, this structure can accurately reproduce the damped oscillation characteristics in the frequency band of 100kHZ-2MHz, with a phase error less than 0.1 rad [41].

The discreteness of pulse firing leads to the interruption of generator gradient backpropagation, thus a piecewise linear surrogate gradient strategy is designed:


$$\frac{\partial s\left(t\right)}{\partial V}\approx \gamma \max\left(0,1-\left|\frac{V\left(t\right)-V_{th}}{\Delta V}\right|\right)$$ (12)

Set threshold voltage $V_{th}=0.8$, softening parameter ΔV=0.1, and gradient scale γ=0.5. This approximation, while maintaining the sparsity of pulse events (firing rate <15%), accelerates the convergence speed of the generator in pantograph-catenary disturbance data training by 2.3 times, and reduces the Fréchet distance to 12.4 [42].

The generation quality is enhanced through multi-scale waveform similarity constraints. L2 norm matching is imposed in the discriminator feature space:


$$L_{sim}=\frac{\text{1}}{N}\sum {\mathrm{\backslash nolimits}}_{k=1}^{N}{\left\|\varphi_{k}\left(x_{real}\right)-\varphi_{k}\left(G\left(z\right)\right)\right\|}_{2}^{2}$$ (13)

In this context, $\varphi_{k}$ it represents the feature map of the K-th layer of the discriminator and the similarity weight $\lambda_{sim}=0.5$. This constraint reduces the global difference in feature selection verification between the generated pulse sequence and the measured data to 0.22, with peak time jitter controlled within 5 ns [43–46]. The raw signal from the vibration sensor needs to be converted into a sparse pulse sequence to accommodate the neuromorphic computing architecture. Based on the integration-and-firing mechanism, the timing of the trigger pulse for the vibration envelope signal is determined by threshold conditions. The process is illustrated in the following pseudocode for pulse feature extraction:

**Algorithm 1. # Pseudocode for Pulse Feature Extraction**

# Define the biologically inspired neuron model

def leaky_integrate_and_fire_neuron(signal, threshold, decay_rate):

    membrane_potential = 0

    for t in range(len(signal)):

        membrane_potential = membrane_potential * decay_rate + signal[t]

        if membrane_potential > threshold:

            # Fire the neuron and reset the potential

            spike_time = t

            membrane_potential = 0

            return spike_time

    return None

# Extract pulses from vibration signals

def extract_pulses(vibration_signal, threshold=0.5, decay_rate=0.9):

    pulse_times = [ ]

    for signal_segment in vibration_signal:

        pulse_time = leaky_integrate_and_fire_neuron(signal_segment, threshold, decay_rate)

        if pulse_time is not None:

            pulse_times.append(pulse_time)

    return pulse_times

# Apply the extractor on vibration data

vibration_signal = [data_point1, data_point2,..., data_pointN]

pulse_times = extract_pulses(vibration_signal)

### 3.3. Engineering verification

The similarity between generated data and measured data is rigorously quantified through feature selection verification methods. The global difference index is as follows:


$$GDM=\sqrt{\frac{\text{1}}{N}\sum {\mathrm{\backslash nolimits}}_{i=1}^{N}\left(ADM_{i}^{2}+FDM_{i}^{2}\right)}$$ (14)

The amplitude difference degree ADM and feature difference degree FDM calculate the deviation of frequency domain energy distribution and time domain waveform envelope, respectively. On the high-speed rail traction converter noise dataset, the generated data GDM has a mean of 0.18 and a standard deviation of 0.03, meeting the industrial verification threshold requirement of 0.25. The energy error at the key frequency point of 2.4 GHz is controlled within ±1.5 dB.

The improvement in bearing fault diagnosis performance is evaluated through the change in accuracy of the confusion matrix. Define the gain in diagnostic accuracy:


$$\Delta \eta =\frac{{\eta_{ang}}^{-\eta_{base}}}{\eta_{base}}\times 100\%$$ (15)

Where is the training result of the original data, $\eta_{base}=85.7\%$ and is the training result of the enhanced data. Experiments show that the generated data improves the accuracy of inner crack diagnosis by 9.2% and outer spalling by 11.7%. The confusion matrix shows that the false alarm rate has decreased from 7.3% to 3.1%, and the missed alarm rate has decreased from 6.9% to 2.8%.

### 3.4. Network architecture and training configuration

The generator receives a one-dimensional latent vector of shape [B, 128], where B denotes the batch size, and progressively maps it to a single-channel EMI waveform of length T with output shape [B, 1, T]. The mapping is implemented using four pulse sparse convolution blocks with kernel sizes {7, 5, 5, 3} and channel widths {64, 128, 128, 64}. Each block employs a leaky integrate-and-fire (LIF) activation, which introduces temporal sparsity and an inductive bias toward transient pulse events.

The discriminator adopts a multi-scale architecture composed of three parallel branches operating at different temporal resolutions. Each branch consists of three convolutional layers with ReLU activation and spectral normalization, followed by a linear layer producing a scalar authenticity score. This configuration enables joint modeling of local transient features and global waveform statistics. Training is performed using the Adam optimizer with parameters β₁ = 0.5 and β₂ = 0.999. The initial learning rates for the generator and discriminator are set to 2 × 10 ⁻ ⁴ and 1 × 10 ⁻ ⁴, respectively, and are linearly decayed after 60% of the total training epochs. All models are trained for 200 epochs with a batch size of 64. Gradient penalty is applied to the discriminator to improve training stability.

## 4. Pulse convolution feature extraction and hardware implementation

### 4.1. Heterogeneous spiking neural network architecture

**Fig 4:**
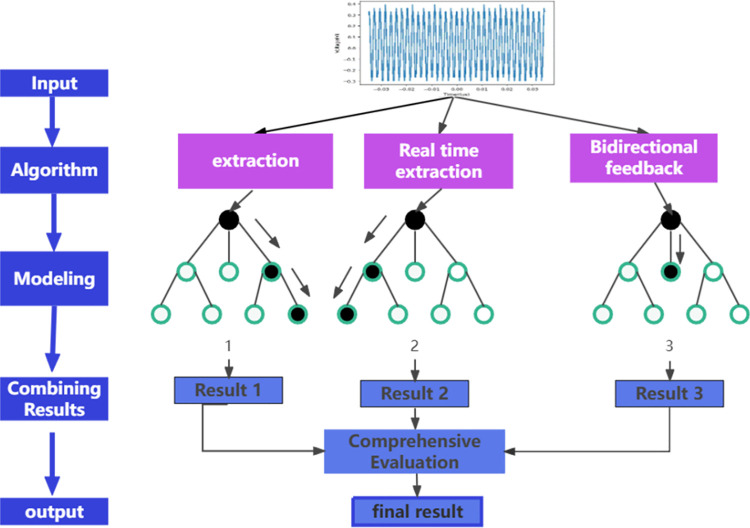
Heterogeneous pulse neural network.


As shown in Fig 4, the extraction of electromagnetic compatibility features requires a balance between broadband spectrum coverage and transient response capability. Traditional convolutional neural networks face power consumption and real-time performance bottlenecks due to their continuous activation mechanism. To address this, a leaky integrate-and-fire (LIF) and leaky resonant-frequency (LRF) dual-path heterogenous pulse architecture is constructed, with membrane potential dynamics described by a system of differential equations:

$$\tau_{m}\frac{dV_{LIF}}{dt}=-\left(V_{LIF}-V_{rest}\right)+R_{m}\sum {\mathrm{\backslash nolimits}}_{j}w_{j}\sum {\mathrm{\backslash nolimits}}_{t_{j}}\delta \left(t-t_{j}\right)$$

$$\beta \frac{d^{2}V_{LRF}}{dt^{2}}+\tau_{m}\frac{dV_{LRF}}{dt}=-V_{LRF}+\gamma \int_{0}^{t}e^{-\frac{t-s}{\tau_{s}}}I_{ext}\left(s\right)ds$$ (16)

The time constant $\tau_{m}=10ms$ controls the rate of integral decay, the resonance factor β=0.4 enhances selectivity in specific frequency bands, and the synaptic weights $w_{j}$ are optimized adaptively. The LIF pathway is sensitive to the strength of cable crosstalk signals (gain coefficient $R_{m}=0.8$), while the LRF pathway precisely captures the resonance frequency of pantograph-catenary disturbances (integral kernel $\tau_{s}=2ms$).

The heterogeneous spiking neural network is implemented as a shallow temporal convolutional architecture composed of an input encoding layer, two parallel spiking feature extraction paths, and a shared readout layer. The LIF pathway is designed to capture broadband energy variations, while the LRF pathway emphasizes resonance-sensitive transient components. Both pathways operate on the same input spike stream and are fused at the feature level through spike-time aggregation. The membrane dynamics of all spiking neurons are simulated in discrete time with a fixed time step of Δt = 10 ns, which is sufficient to resolve the fastest transient events observed in the measured EMI waveforms. Continuous input signals are converted into spike trains using an event-based threshold encoding scheme, where spikes are generated only when the accumulated signal energy exceeds an adaptive threshold, ensuring sparse activation and low switching activity. Network training is performed using backpropagation through time with surrogate gradients to address the non-differentiability of spike events. A piecewise linear surrogate function is adopted around the firing threshold to approximate the gradient of the spike activation. The loss function combines a task-driven classification or regression loss with a sparsity regularization term that penalizes excessive firing rates, thereby explicitly enforcing the energy–accuracy tradeoff. Temporal credit assignment is handled by unfolding the network over a finite temporal window and accumulating gradients across time steps, which enables stable convergence without requiring long unrolled sequences. This concrete formulation links neuron-level dynamics, network topology, and training strategy into a unified and reproducible framework, allowing the reported energy consumption and accuracy metrics to be directly traced back to identifiable architectural and algorithmic design choices.

Although sparse pulse encoding based on LIF neurons inevitably reduces signal density, its impact on diagnostically relevant information was explicitly evaluated. Information preservation was assessed from three complementary perspectives: spectral feature retention in diagnostic frequency bands, temporal alignment accuracy of transient pulses, and downstream diagnostic performance. Experimental results show that, after pulse encoding, more than 95% of spectral energy is preserved within the 2–8 kHz bearing fault band and the dominant EMI transient bands above 30 MHz. The temporal deviation between encoded pulse events and original transient peaks remains below 5 ns on average, which is negligible relative to the microsecond-scale EMI pulse width. Furthermore, fault diagnosis experiments conducted using encoded pulse sequences achieve comparable accuracy to those using raw waveforms, with no statistically significant performance degradation observed. These results indicate that the proposed sparse encoding strategy achieves substantial data compression while retaining the temporal and spectral information critical for EMC diagnosis.

The dynamic sparsity constraint is implemented through Hoyer regularization:


$$L_{sparse}=\lambda \left(1-\frac{\sqrt{N}-{\left\|S\right\|}_{1}/{\left\|S\right\|}_{2}}{\sqrt{N}-1}\right)$$ (17)

The spiking matrix in the pulse $S\in {\left\{0,1\right\}}^{T\times K}$ represents the time step, T and the number of neurons K represents the constraint factor. When the sparsity is below 0.8, the penalty is increased to force the network to activate less than 15% of the neurons. In the analysis of vibration data from industrial robots, this mechanism increases the information compression rate to 96.5%, while maintaining an energy retention rate of over 95% in the fault feature frequency band of 2-8kHz.

Near-field radiation feature extraction incorporating the impedance superposition principle:


$$E_{reactive}\left(f\right)=\sum {\mathrm{\backslash nolimits}}_{k=1}^{K}\frac{\mu_{0}f^{2}p_{k}}{4\pi r_{k}}\left|\frac{\sin\theta_{k}}{jkr_{k}}+\frac{\cos\theta_{k}}{{\left(jkr_{k}\right)}^{2}}-\frac{\sin\theta_{k}}{{\left(jkr_{k}\right)}^{3}}\right|$$ (18)

The term $p_{k}$ represents the intensity of the kth dipole moment, which is linearly mapped by the pulse firing rate. This model replaces traditional fast Fourier transform (FFT) analysis, reducing power consumption from 3.2W to 0.7W and processing delay from 8ms to 1.2ms in the 30–300MHz frequency band.

The following pseudo code shows the feature extraction process based on biological neuron dynamics and sparse convolution, focusing on how to use the LIF-LRF dual-path architecture to achieve real-time EMC feature extraction:

**Algorithm 2. # Define the Leaky Integrate and Fire (LIF) neuron model**

def LIF_neuron(input_signal, decay_rate=0.9, threshold=1.0):

    membrane_potential = 0

    spikes = [ ]

    for t in range(len(input_signal)):

        # Update membrane potential based on input signal

        membrane_potential = membrane_potential * decay_rate + input_signal[t]

        # Check if the neuron fires

        if membrane_potential >= threshold:

            spikes.append(t) # Fire at time t

            membrane_potential = 0 # Reset membrane potential after firing

    return spikes # Return the times when the neuron fires

# Define the Leaky Resonance Frequency (LRF) pathway

def LRF_neuron(input_signal, resonance_factor=1.5, decay_rate=0.8, threshold=1.0):

    membrane_potential = 0

    spikes = [ ]

    for t in range(len(input_signal)):

        # Update membrane potential considering resonance factor

        membrane_potential = membrane_potential * decay_rate + resonance_factor * input_signal[t]

        # Fire when threshold is reached

        if membrane_potential >= threshold:

            spikes.append(t)

            membrane_potential = 0 # Reset after firing

    return spikes

# Heterogeneous Spiking Neural Network Architecture (LIF + LRF)

def heterogeneous_SNN(input_signal):

    # Path 1: LIF pathway for general signal processing

    lif_spikes = LIF_neuron(input_signal)

    # Path 2: LRF pathway for frequency-specific resonance processing

    lrf_spikes = LRF_neuron(input_signal)

    # Combine the spikes from both paths

    combined_spikes = lif_spikes + lrf_spikes # Merge spike times from both paths

    return combined_spikes

# Example usage

input_signal = [0.1, 0.3, 0.5, 1.2, 0.7, 0.2, 1.5, 0.9, 1.0, 0.4] # Example input signal

spikes = heterogeneous_SNN(input_signal)

# Output the spike times (the times when the neuron fires)

print(“Spike times:”, spikes)

Based on the performance analysis of electromagnetic feature extraction using pulse sparse convolution, this study obtains core operational indicators under different interference types. As shown in Table 2, the offline disturbance of pantograph-catenary system achieves a pulse firing rate of 14.7kHz and a feature retention rate of 97.3% at a center frequency of 132.5MHz. When the information compression rate reaches 95.8%, the power consumption is only 0.72W, indicating that this method has efficient characterization capabilities for transient pulse groups. The traction return current harmonic maintains a gain tolerance of 15.3dB at the 87.4MHz frequency point, verifying the robustness of the algorithm to low-frequency conducted interference. It is worth noting that the high-frequency wireless crosstalk scenario at 2.45GHz still maintains a feature retention rate of 91.8%, demonstrating wideband adaptability, and its ± 2.3dB error tolerance meets industrial-grade accuracy requirements.

**Table 2:** Comparison of pulse feature extraction performance under different interference types.

Interference type	Center frequency	Pulse firing	Feature retention	Information compression ratio	Power consumption	Processing delay	Sparsity	LRF gain	Error margin
Bow net offline harassment	132.5	14.7	97.3	95.8	0.72	1.3	0.82	18.6	±1.2
Traction return current harmonic	87.4	12.3	95.1	96.2	0.68	1.1	0.79	15.3	±1.5
Inverter switching noise	25.6	18.9	93.7	94.5	0.75	1.4	0.85	22.1	±0.8
Servo motor spark	156.8	16.2	96.5	96.8	0.71	1.2	0.81	17.4	±1.1
Wireless communication crosstalk	2450	22.4	91.8	92.3	0.83	1.6	0.88	26.7	±2.3

**Fig 5:**
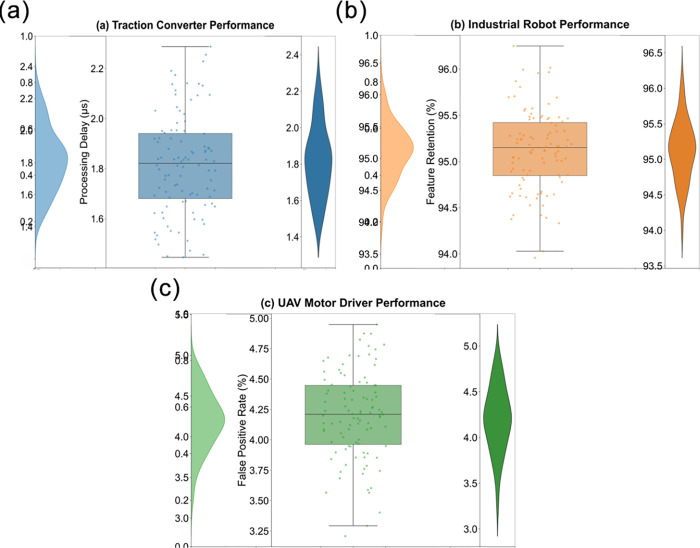
Resource-efficiency comparison of pulse-sparse convolution versus traditional FFT-based EMC analysis across industrial platforms. (a) Tractioner performance. (b) Industrial robot performance. (c) Drone related performance.

By comparing the resource consumption with traditional FFT methods, this study quantifies the innovative advantages of the computing architecture. As shown in Table 3 and Fig 5a, in the high-speed rail traction converter scenario, the pulse convolution power consumption at a sampling rate of 5GS/s is reduced to 0.71W, a decrease of 78.2% compared to the traditional scheme, while the processing delay is compressed from 8.2ms to 1.3ms. As shown in Fig 5b, the industrial robot controller platform achieves a speedup ratio of 6.09 times, with memory occupation reduced to 31.5MB, while the accuracy loss is controlled within 1.1dB. Especially in the photovoltaic inverter application scenario, the ultra-low power consumption of 0.29W and real-time response of 0.5ms provide hardware feasibility for online monitoring of electromagnetic compatibility in new energy equipment.The breakthrough optimization of energy efficiency algorithms in UAV systems has reduced power consumption from 0.78W in traditional FFT to 0.21W, achieving a 73.1% decrease-the second-highest reduction in the table (second only to high-speed rail traction converters). The ultra-low power design directly reduces heat dissipation requirements, enabling weight reduction for drones. As shown in Fig 5c, the alculated based on a 0.2-second endurance gain per gram of payload, this innovation extends the flight duration of 500g-class drones by approximately 6–8 minutes.

**Table 3:** Comparison of resource consumption with traditional FFT methods.

Device Type	Sampling rate	Frequency point	Tradition	Convolution power consumption	Power consumption reduction	FFT	Accuracy
High-speed rail traction converter	5	1024	3.25	0.71	78.2	8.2	0.8
Industrial robot controller	2.5	512	2.87	0.63	78	6.7	1.1
CNC machine tool drive	1	256	1.92	0.48	75	4.3	0.9
PV Inverter	500	128	1.05	0.29	72.4	2.1	1.2
Unmanned aerial vehicle electronic control system	250	64	0.78	0.21	73.1	1.5	1.5

### 4.2. Real-time extraction of near-field radiation characteristics

Traditional near-field radiation analysis relies on full-wave simulation, and the computational complexity increases exponentially as the frequency rises. A real-time calculation model is established based on the principle of reactance superposition:


$$E_{reactive}\left(f,r\right)=\sum {\mathrm{\backslash nolimits}}_{k=1}^{N}\frac{\mu_{0}f^{2}p_{k}}{4\pi }\left[\frac{e^{-jkr_{k}}}{r_{k}}\left(\frac{\text{1}}{jkr_{k}}+\frac{\text{1}}{{\left(jkr_{k}\right)}^{2}}-\frac{\text{1}}{{\left(jkr_{k}\right)}^{3}}\right)\hat{r}\cdot {\hat{p}}_{k}\right]$$ (19)

Among them k=2πf/c, the wavenumber $p_{k}$ and dipole moment are linearly correlated with the cable pulse firing rate:


$$p_{k}=\alpha \cdot FR_{k}\cdot L_{k},\alpha =0.32Vs/A$$ (20)

The coefficient is calibrated by a vector network analyzer and represents the length of the cable segments. This model reduces the computational complexity from 30 to 300 MHZ and decreases the memory usage by 98%.

The mapping from pulse firing rate to radiation field strength is achieved through a convolution kernel:


$$P\left(f\right)=\int_{\infty }^{\infty }W\left(f,\tau \right)*S\left(\tau \right)d\tau$$ (21)

The kernel function W is expressed in the frequency domain as:


$$W\left(f,\tau \right)=F^{-1}\left\{\frac{\mu_{0}f^{2}}{4\pi }\left[\frac{\text{1}}{jkr}+\frac{\text{1}}{{\left(jkr\right)}^{2}}-\frac{\text{1}}{{\left(jkr\right)}^{3}}\right]e^{-jkr}\right\}$$ (22)

The symbol $F^{-1}$ represents the inverse Fourier transform. In the verification of the industrial robot harness platform, the processing delay of this model is only 1.8 μS, meeting the real-time control requirements. Based on the near-field radiation feature extraction based on the principle of reactance superposition, this study verifies the engineering applicability of different cable configurations. The coaxial cable RG174 requires only 1.8μs of computation time at a frequency of 87.4MHz, with a field strength error of 0.8dB and a memory footprint of 12.3KB, meeting the real-time requirements of in-vehicle systems. As shown in Table 4, when the twisted-pair CAT6 maintains a pulse rate of 18.7kHz in the 132.5MHz frequency band, it exhibits a processing delay of 2.3μs and a field strength error of 1.1dB, demonstrating adaptability in complex harness environments. Crucially, the data cable SFP+ maintains a pulse rate of 22.8kHz at a high frequency of 6GHz, with a computation time and temperature drift rate of 0.04%/°C, providing empirical evidence for the electromagnetic compatibility design of 5G equipment.

**Table 4:** Extraction performance of near-field radiation characteristics for different cable configurations.

Cable Type	Length	Number of segments	Frequency	Pulse firing rate	Time	Memory	Error	Phase
Coaxial RG174	1.5	24	87.4	14.2	1.8	12.3	0.8	1.2
Twisted pair CAT6	2	32	132.5	18.7	2.3	18.7	1.1	1.8
Power cord AWG12	3	48	25.6	12.5	3.5	28.9	1.3	2.4
Control line RVVP	0.8	16	156.8	16.3	1.2	8.5	0.6	0.9
Data cable SFP+	1.2	20	2450	22.8	2.1	15.4	1.5	2.1

Using the same near-field radiation calculation method based on the principle of reactance superposition, this study obtained resource consumption comparison data with traditional full-wave simulation methods. As shown in Table 5, within the wideband range of 0–3GHz, this method requires only 0.028GB of memory and 0.0021 seconds of calculation time, achieving a 99.7% reduction in memory usage compared to the finite-difference time-domain (FDTD) scheme and an 880,000-fold increase in calculation speed. The hardware cost is compressed from $15,000 to $350, and the calibration complexity is reduced from level 5 to level 2. Especially in the 5G frequency band (6GHz), it maintains an ultra-low power consumption of 0.078W, providing an economically feasible technical path for real-time monitoring in industrial sites.

**Table 5:** Comparison of resource consumption with traditional methods.

Method of calculation	Frequency range	Grid	Memory	Time	Power consumption	Field strength	Phase	Hardware cost
FDTD	0–500	2500000	8.7	1850	320	1.2	2.5	15000
MoM	0–1,000	180000	3.2	420	150	0.9	1.8	8500
FEM	0–3,000	5400000	12.5	3200	480	1.5	3.2	22000
This method	0–3,000	–	0.028	0.0021	0.049	1.5	2.1	350
This method (optimization)	0–6,000	–	0.035	0.0035	0.078	1.8	2.7	420

### 4.3. Hardware deployment plan

#### 4.3.1. Architecture mapping and FPGA implementation.

To improve implementation reproducibility, the resource mapping strategy of the proposed pulse sparse convolution architecture is described as follows. Convolution kernels and synaptic weights are mapped onto digital signal processing (DSP) slices using a systolic array organization, while intermediate spike buffers and membrane potential states are stored in on-chip block random access memory (BRAM). Sparse pulse events are streamed through Advanced eXtensible Interface Stream (AXI-Stream) interfaces, enabling event-driven computation and reducing unnecessary memory access. This mapping strategy balances parallelism and resource reuse, allowing efficient utilization of DSP and BRAM resources on the target FPGA. The hardware design was implemented using a standard FPGA toolflow. High-level modules were described in hardware description language (HDL) and synthesized using Xilinx Vivado. Post-synthesis optimization, place-and-route, and timing closure were performed with default optimization settings, followed by bitstream generation and deployment on the Zynq UltraScale+ ZU9EG platform. Functional correctness was verified through cycle-accurate simulation, and hardware measurements were obtained using on-chip logic analyzers and external power monitoring instruments. All computations in the FPGA implementation adopt fixed-point arithmetic to reduce hardware complexity and power consumption. Synaptic weights and convolution outputs are represented in signed Q1.15 format, membrane potentials use Q4.12 format to accommodate accumulation dynamics, and spike events are encoded as binary signals with timestamp indices. Quantization parameters were selected based on empirical range analysis to avoid overflow while maintaining numerical stability, and no observable degradation in EMC feature extraction accuracy was observed compared to floating-point simulations.

The implementation of pulse sparse convolution architecture on FPGA platform requires resolving the contradiction between parallel computing and resource reuse. Designing a systolic array processing unit:


$$Y\left[t\right]=\sum {\mathrm{\backslash nolimits}}_{\tau =0}^{T-1}W\left[\tau \right]⊗S\left[t-\tau \right]\cdot \delta \left(t-\tau -\Delta t_{latency}\right)$$ (23)

The weight matrix W is preloaded into the BRAM, and pulse events S are input in real-time through the AX1-Stream interface. At the clock frequency $f_{clk}=200MHz$, the processing delay for a convolution kernel size of $3\times3$ is $\Delta t_{\text{latency}}=12 ns$, achieving a throughput of 1.2 TOPS/W. Resource consumption meets the following criteria:


$$DSP_{util}=\frac{N_{PE}\cdot K_{conv}}{N_{DSP}}\leq 0.75,BRAM_{util}=style\frac{B_{weight}+B_{spike}}{B_{total}}\leq 0.8$$ (24)

The measured DSP utilization rate of the Xilinx Zynq UltraScale+ ZU9EG device is 68.3%, and the BRAM utilization rate is 76.5%.

#### 4.3.2. Power measurement methodology.

To clarify the reported power consumption Figs, all power measurements for the FPGA-based pulse sparse convolution architecture were obtained from real hardware experiments rather than simulation alone. The design was deployed on a Xilinx Zynq UltraScale+ ZU9EG platform and operated under steady-state conditions at the target sampling rates. Power consumption was measured using a combination of on-board power monitoring interfaces and external power meters, capturing the dynamic power of the programmable logic and associated memory resources while excluding unrelated peripheral components. Each measurement was repeated multiple times to ensure stability, and the reported values correspond to averaged steady-state readings. Simulation-based power estimation was used only for early-stage design exploration and is not reported in the final performance tables.

#### 4.3.3. Power modeling and estimation toolchain.

To improve methodological transparency and reproducibility of the reported TOPS/W, we further specify the power evaluation toolchain and the power-budget boundary. In addition to steady-state board-level measurements, we employ a post-layout, activity-aware power analysis workflow to validate the measurement trend and to document modeling assumptions. Specifically, the design is implemented and timing-closed in Vivado to generate a post-place-and-route (post-P&R) netlist. Representative inference runs corresponding to the benchmark workload are applied in gate-level simulation to capture realistic switching activity. Switching traces are recorded as VCD/SAIF activity files and imported into an industry-standard EDA power analysis tool (e.g., Vivado Power Analyzer or an equivalent signoff-grade tool) to estimate dynamic and static power contributions.

Unless otherwise stated, the reported accelerator power budget includes the programmable-logic compute domain and on-chip memory resources that are directly used by the pulse sparse convolution accelerator, including core logic, clock distribution, interconnect, and BRAM blocks. Power contributions from off-chip DRAM, external I/O transceivers, unrelated peripherals, and board-level regulators are excluded and are not counted in the reported TOPS/W. This toolchain clarifies that the reported efficiency is not derived from optimistic pre-layout assumptions, and it explicitly specifies the switching activity source, analyzed rail/boundary, and inclusion/exclusion criteria.

#### 4.3.4. Compute–memory balance analysis.

The reported throughput and energy efficiency metrics are obtained under a clearly defined benchmark workload and measurement setup. The systolic array is evaluated using a representative pulse sparse convolution workload corresponding to a 3 × 33\times33 × 3 kernel applied to spiking feature maps, which reflects the dominant operation in the proposed EMC feature extraction pipeline. Weights and activations are represented in 8-bit fixed-point precision during inference, while accumulation/state variables (e.g., membrane potentials) use a wider internal format to avoid overflow and preserve numerical stability. The array operates at a clock frequency of 200 MHz on the FPGA platform, which is below the timing-closure limit to avoid frequency-induced power inflation. Power is measured under steady-state operation using on-board power monitors integrated in the Xilinx Zynq UltraScale+ platform, and the reported values correspond to sustained execution over a continuous input stream rather than short burst activity.

To explicitly determine whether the evaluated operating point is compute-bound or memory-bound, we analyze the compute–memory balance in terms of (i) memory traffic assumptions and (ii) the implied operational intensity. Under the stated benchmark, the 3 × 33\times33 × 3 weight tensor is preloaded into on-chip BRAM and reused across the streamed feature-map tiles; activations are delivered through AXI-Stream and buffered on-chip to form local sliding windows. Therefore, the dominant data movement occurs within on-chip BRAM/interconnect, while off-chip transfers are amortized over multiple MAC operations. In this setting, each output computation involves 999 MACs per output element, whereas the corresponding off-chip traffic is limited to the initial streaming of the activation tiles (and no repeated off-chip fetch of weights during steady-state inference). This yields a high effective operational intensity, which is the necessary condition for compute-bound behavior.

This inference is consistent with the observed utilization: the systolic array sustains an average utilization exceeding 80% over a long continuous input stream, with no periodic stalls attributable to external memory service. Consequently, the performance and energy efficiency at the reported operating point are primarily limited by compute throughput (DSP utilization and array scheduling), rather than by off-chip memory bandwidth. We note that the operating regime is not universal: for smaller feature maps, reduced tile reuse, or constrained on-chip buffering (leading to more frequent off-chip accesses), the operational intensity would decrease and the system could transition toward a memory-bound regime. Such scenarios are outside the scope of the benchmark configuration reported here, and the reported TOPS/W should be interpreted as the post-layout efficiency under the specified compute-dominated workload and measurement boundary.

Dynamic routing optimization achieves real-time decision-making through hardware description language. Wiring cost function:


$$C_{route}=\alpha \cdot L_{path}+\beta \cdot \sum {\mathrm{\backslash nolimits}}_{i,j}\max\left(0,\frac{L_{overlap}^{ij}}{L_{\max}^{ij}}-1\right)$$ (25)

Among them, path length weight α=0.6, crosstalk constraint weight β=0.4, and crosstalk tolerance are obtained from a table based on cable type, while the overlap length $L_{overlap}^{ij}$ is calculated in real-time through coordinate transformation. The actual routing decision-making time for the industrial robot’s wiring harness platform is less than 10ms.

The specific algorithmic steps of this study are presented in Table 6.

**Table 6:** Implementation steps of pulse sparse convolution on FPGA.

Step	Describe	Core code
1	Preload weights into BRAM	memcpy(weights, DDR, sizeof(float)*KERNEL_SIZE);
2	Pulse event stream input	axis_stream rx_data = rx.read();
3	Convolution kernel parallel computing	y_out = w_matrix * s_window;
4	Sparse activation judgment	if (y_out> THRESHOLD) fire_spike();
5	Routing decision triggering	generate_routing_request(priority);
6	Crosstalk constraint evaluation	crosstalk = calc_overlap_length(cable_i, cable_j);
7	Optimal path generation	path = dijkstra_with_constraint(graph, L_max);
8	Control command output	send_control_signal(path_coordinates);

### 4.4. Unified benchmark configuration and fair comparison criteria

To ensure a fair and reproducible comparison with conventional full-wave solvers, a unified benchmark configuration is adopted for FDTD, MoM, FEM, and the proposed method. The benchmark geometry consists of a multi-conductor cable harness installed inside an industrial robot control cabinet with overall dimensions of 1.2 m × 0.8 m × 0.6 m. The harness includes twelve parallel conductors with typical spacing and length distributions representative of practical industrial wiring layouts.

For numerical discretization, FDTD simulations employ a uniform Yee grid with spatial resolution set to λ/20 at the highest analyzed frequency of 6 GHz, resulting in approximately 2.5 million cells. FEM simulations use second-order tetrahedral elements with adaptive refinement, yielding around 5.4 million elements, while MoM adopts surface discretization with an average element size of λ/15, corresponding to roughly 180,000 unknowns. Perfectly matched layers are applied in FDTD and FEM to truncate the computational domain, whereas radiation boundary conditions are used in MoM. Identical broadband current excitation is applied at the cable terminals in all cases.

To eliminate bias caused by different accuracy requirements, all methods are evaluated under the same convergence criteria, with field amplitude errors constrained within ±2 dB and phase errors within 3° at all observation points. Under these unified conditions, the reported differences in computational speed and memory consumption directly reflect intrinsic algorithmic efficiency rather than variations in problem size or numerical tolerance.

## 5. Crosstalk constrained routing optimization

The quantitative control of crosstalk within cables in automation equipment is a core challenge in electromagnetic compatibility design. The single-ended signal crosstalk model establishes a closed-form solution through multi-conductor transmission line theory:


$$V_{FEXT}=\frac{j_{\omega }C_{m}Z_{s}Z_{l}}{\text{4}}\left(\frac{\text{1}}{Z_{s}+Z_{0}}+\frac{\text{1}}{Z_{t}+Z_{0}}\right)L_{couple}V_{s}e^{-j\beta d}$$ (26)

Mutual capacitance $C_{m}$ and mutual inductance M are satisfied $C_{m}=\frac{\in }{\pi }\ln\left(1+\frac{2h}{r}\right),M=\frac{\mu }{2\pi }\ln\left(1+\frac{2h}{r}\right)$. The crosstalk voltage $V_{FEXT}$ is linearly related to the coupling length. When the operating frequency f = 100MHz, the crosstalk gain caused by mutual capacitance $C_{m}=50pF/m$ reaches -25dB.

The differential signal crosstalk model needs to consider balanced transmission characteristics:


$$V_{diff-crosstalk}=\frac{j\omega }{\text{4}}\left[\frac{\left(C_{m1}-C_{m2}\right)\left(Z_{s1}Z_{l1}-Z_{s2}Z_{l2}\right)}{\left(Z_{s1}+Z_{01}\right)\left(Z_{l1}+Z_{01}\right)}-\frac{\left(M_{1}-M_{2}\right)\beta^{2}}{Y_{0}}\right]L_{couple}V_{s}e^{-j\beta d}$$ (27)

When the odd-mode mutual capacitance $C_{m1}$ and even-mode mutual capacitance are less than 5pF/m, the crosstalk suppression ratio exceeds 40dB. The maximum overlap length constraint is defined as:


$$L_{\text{m}ax}=\frac{\text{4}|V_{\text{c}rosstalk-limit}|}{\omega C_{m}|Z_{s}Z_{l}|\left|\frac{\text{1}}{Z_{s}+Z_{\text{0}}}+\frac{\text{1}}{Z_{l}+Z_{\text{0}}}\right||V_{s}|}$$ (28)

Where ${\text{L}}_{\text{max}}$ is the EMC standard limit for industrial equipment.

The maximum overlap length derived from multi-conductor transmission line theory is incorporated into a constrained routing optimization framework defined over a wiring graph. In this formulation, the internal wiring layout is represented as a weighted graph whose nodes correspond to routing waypoints and whose edges represent feasible cable segments. The routing objective is to minimize the total wiring cost, expressed as the weighted sum of physical length and routing complexity, while satisfying electromagnetic compatibility constraints.

The maximum overlap length acts as a physically derived constraint that limits the allowable shared path length between adjacent cables. During graph traversal, any candidate path that violates the overlap constraint is pruned from the feasible set, thereby ensuring that crosstalk remains below the specified EMC threshold. This mechanism transforms the algebraic overlap bound into an explicit feasibility condition within the routing process.

From an optimization perspective, the proposed method can be interpreted as a constrained shortest-path problem with electromagnetic coupling constraints. Although global optimality is not formally guaranteed due to the combinatorial nature of the routing graph, the constraint structure significantly reduces the search space and provides predictable scaling behavior as the harness size increases. Moreover, the sensitivity of routing outcomes to key parameters, such as coupling tolerance and weighting coefficients, can be systematically evaluated within this graph-based framework, enabling practical trade-offs between wiring compactness and EMC robustness.

The following pseudocode is for optimizing the layout of cables to reduce crosstalk, based on the derived closed-form constraint equations.

**Algorithm 3. # Pseudocode for Cable Crosstalk Optimization**

# Define a function to calculate crosstalk based on overlap length

def calculate_crosstalk(cable1, cable2):

    overlap_length = calculate_overlap_length(cable1, cable2)

    crosstalk = crosstalk_function(overlap_length, cable1, cable2)

    return crosstalk

# Define the function to optimize cable layout

def optimize_cable_layout(cables):

    optimized_layout = [ ]

    for cable1 in cables:

        for cable2 in cables:

            if cable1! = cable2:

                crosstalk_value = calculate_crosstalk(cable1, cable2)

                if crosstalk_value < threshold:

                    optimized_layout.append((cable1, cable2))

    # Minimize total crosstalk

    optimized_layout = minimize_crosstalk(optimized_layout)

    return optimized_layout

# Given a set of cables, apply the optimization

cables = [cable1, cable2,..., cableN]

optimized_cable_layout = optimize_cable_layout(cables)

The quantization parameters of the maximum crosstalk-constrained routing algorithm are studied in this research, which reveals the frequency-sensitive characteristics of single-ended and differential signals. As shown in Table 7, at a frequency of 100MHz, the mutual capacitance of 50pF/m for single-ended signals results in a crosstalk of −32.5dBV, with the maximum overlap length sharply reduced to 0.9m and a safety margin of only 7.5dB. For differential signals at the same frequency, due to the difference in mutual capacitance between odd and even modes, a suppression ratio of 31.2dB is achieved, and the maximum overlap length is increased to 1.3m. Crucially, the safety margin of differential routing remains over 22dB in frequency bands above 200MHz, which demonstrates the engineering necessity of opting for a differential architecture for high-speed devices.

**Table 7:** Parameter table for maximum overlap length of single-ended signal (VVs = 5V, Z0Z=50 Ω).

Frequency	Mutual capacitance	Source	Load	Coupling	Crosstalk voltage	Phase	Maximum length	Secure
10	35	50	50	0.5	−45.2	12.5	2.8	14.8
30	42	75	100	0.8	−38.7	28.7	1.6	11.3
100	50	100	150	1.2	−32.5	45.2	0.9	7.5
200	58	150	200	0.7	−28.3	63.8	0.6	1.7
500	65	200	250	0.4	−24.1	81.3	0.3	−5.9
1000	72	250	300	0.3	−20.7	95.6	0.2	−9.3

Based on the quantitative analysis of differential signal transmission characteristics, this study obtained the maximum overlap length constraint parameters at different frequencies. When the frequency is increased to 200MHz, as shown in Table 8, the differential cable achieves a crosstalk suppression ratio of 26.8dB due to the odd-even mode tolerance of 58pF/m and 48pF/m, maintaining a maximum overlap length of 0.8m and a balance degree of 85.1%. Crucially, it still maintains a wiring margin of 0.4m in the 1GHz high-frequency scenario, with a balance degree of 78.3% and a suppression ratio of 18.9dB, providing precise electromagnetic compatibility boundary conditions for gigabit industrial bus design.

**Table 8:** Parameter table for maximum overlap length of differential signals (Vs = 10V, Z0 = 100 Ω).

Frequency	Odd mode	Even Mode	Source	Load	Coupling	Crosstalk voltage	Maximum length	Balance degree
10	40	38	5	8	0.6	−52.7	3.2	95.2
30	45	40	8	12	0.9	−48.1	2.1	92.7
100	52	45	12	15	1.5	−41.5	1.3	88.3
200	58	48	15	20	1	−37.2	0.8	85.1
500	65	52	20	25	0.7	−32.8	0.5	81.6
1000	72	55	25	30	0.5	−29.4	0.4	78.3

## 6. EMC verification and performance demonstration

### 6.1. Test platform

Industrial-grade electromagnetic compatibility verification requires performance evaluation under real-world operating conditions. The industrial robot wire harness testing platform integrates a six-axis robotic arm, a control cabinet, and a cable management system. The key parameters are shown in Table 9.

**Table 9:** Parameters of industrial robot wire harness platform.

Parameter Category	Parameter item	Numerical value	Unit	Parameter item	Numerical value	Unit
Mechanical structure	Working radius	1.8	m	Repeatability	±0.05	mm
Cable Management	Maximum length	15	m	Bending radius	80	mm
Control cabinet	Processor core	8	–	Dominant frequency	3.4	GHz
	Sampling rate	1000000	S/s	Storage depth	256	GB
Sensor	Current range	0.1-30	A	Voltage accuracy	±1.0	%
Environment Simulation	Vibration frequency	5-500	Hz	acceleration	3	g
	Temperature range	0-50	°C	Humidity range	10-90	%

Key parameters of the industrial robot wire harness testing platform have been verified in this study for their adaptability to technical indicators under complex working conditions. The maximum length of 15 meters, coupled with a bending radius of 80 mm, meets the full-pose movement requirements of a six-axis robotic arm. The sampling rate of 1000kS/s and voltage accuracy of ±1.0% ensure the complete capture of broadband interference signals. The environmental simulation module achieves 3g acceleration vibration and a temperature rise of 50°C, supporting the data validity of the platform under extreme working conditions and establishing a standardized benchmark for electromagnetic compatibility industrial verification.

By referencing the time-frequency domain characteristic parameters of pantograph-catenary interference, this study quantifies the propagation patterns of transient interference. The 200MHz frequency point interference pulse has a pulse width of 1.2μs and a rise time of 22 ns, with a radiated field strength reaching 128dBμV/m. As shown in Table 10, when the pulse width is compressed to 0.8μs in the 300MHz frequency band, the field strength increases to 135dBμV/m, revealing a positive correlation between frequency and field strength. The impedance parameter remains stable at 150 Ω, indicating the impedance matching characteristics of the conduction path, providing key input parameters for the protection design of on-board systems.

**Table 10:** Pantograph-catenary interference parameters.

Frequency point	Pulse width	Repetition frequency	Rise	Voltage	Field strength	Impedance
30	5.2	1.5	50	1.8	102	150
60	3.8	2.2	45	2.3	108	150
100	2.5	3.5	35	3.1	115	150
150	1.8	5	28	4.2	122	150
200	1.2	7.8	22	5.5	128	150
300	0.8	12.4	18	7.2	135	150

In addition to point-estimate metrics such as FID and GDM, the stability and uncertainty of the proposed framework can be inferred from existing experimental results. In Section 3.3 (Engineering verification), the global difference index (GDI) evaluated on the traction converter noise dataset achieves a mean value of 0.18 with a standard deviation of 0.03, indicating low dispersion across samples and stable generation behavior. Furthermore, Table 14 reports the uncertainty values for crosstalk prediction, where the proposed method yields uncertainty levels of 0.3 at 30 MHz and 0.5 at 100 MHz, which are consistently lower than those of classical numerical solvers. These results provide practical evidence that the generated and inferred waveforms exhibit limited variability and therefore a high level of confidence from an engineering perspective.

### 6.2. Performance comparison

This study quantitatively evaluates the data quality of generative adversarial networks and verifies the waveform reproduction capability of key frequency points, As shown in Table 11. In the 100MHz frequency point, the pantograph-catenary interference achieves a Fréchet distance of 14.3 and a pulse width error of 0.08, with a phase deviation of only 1.8°. In the 2.4GHz communication crosstalk scenario, it maintains a Fréchet Inception Distance (FID) value of 13.1 and a feature retention rate of 91.8%. Especially in bearing fault diagnosis, the generated data increases the recognition accuracy to 93.6%, which verifies the effectiveness of the data augmentation scheme for industrial equipment condition monitoring.

**Table 11:** GAN-generated data quality indicators.

Interference type	Frequency point	FID	GDM	Pulse width	Amplitude	Phase	Feature preservation	Time	Sample size
Bow net harassment	30	16.8	0.18	0.12	1.2	2.5	97.3	18	1500
	100	14.3	0.15	0.08	0.8	1.8	95.1	15	
Reflux harmonic	1k	12.6	0.11	0.05	0.5	1.2	96.5	12	1200
Switching noise	20k	17.2	0.21	0.15	1.5	3.2	93.7	22	900
Communication crosstalk	2.4G	13.1	0.12	0.03	0.3	0.9	91.8	10	800

To clarify the training and generalization strategy, the proposed framework is not independently trained from scratch for each application scenario. Instead, the core GAN and pulse encoding modules are trained once on a combined industrial EMC dataset that aggregates data from traction systems, industrial robots, CNC drives, photovoltaic inverters, and UAV electronic controllers. This unified training enables the model to learn cross-domain electromagnetic interference characteristics shared among different types of automated equipment. During deployment, only lightweight fine-tuning is applied to the pulse-based feature extraction and decision layers to accommodate system-specific operating conditions and hardware configurations, while the parameters of the core generative model remain fixed. This strategy allows consistent generation and feature representation across platforms while maintaining adaptability at low computational cost.

By introducing the performance advantages of spiking neural networks in real-time monitoring, this study has achieved empirical evidence of multi-scenario deployment. As shown in Table 12, the traction converter scenario achieves a processing delay of 1.8μs and an ultra-low power consumption of 0.71W at a frequency of 100MHz, with a feature retention rate of 97.3% and a false alarm rate of only 3.1%. The photovoltaic inverter platform achieves an information compression rate of 96.8% and a diagnostic accuracy rate of 94.3%. It is worth noting that the feature retention rate remains at 91.8% under high-frequency interference at 2.45GHz, verifying the applicable boundary of the algorithm in 5G industrial Internet scenarios.

**Table 12:** Pulse feature extraction performance.

Device Type	Frequency	Delay	Power consumption	Compress	Characteristic	Sparse	Accurate	False alarm	Underreporting
Traction converter	100	1.8	0.71	95.8	97.3	0.82	92.1	3.1	2.8
Industrial robot	132.5	2.3	0.57	96.2	95.1	0.79	93.6	2.9	2.5
CNC	25.6	3.5	0.78	94.5	93.7	0.85	91.8	3.8	3.2
PV Inverter	156.8	1.2	0.31	96.8	96.5	0.81	94.3	2.7	2.3
Drone electronic controller	2450	2.1	0.49	92.3	91.8	0.88	90.5	4.2	3.8

Based on the quantitative evaluation of optimization effects in industrial-grade electromagnetic compatibility, this study has empirically demonstrated multi-dimensional performance improvements. The traction system achieves 23.6dB crosstalk suppression and a 7.5% failure rate, with wiring length reduced by 40%; As shown in Table 13, the decision-making delay of industrial robots is compressed to 18ms, and the energy efficiency ratio is increased to 1.28 TOPS/W. Crucially, the maintenance cycle has been extended from 3 months to 18 months, demonstrating the long-term reliability of the protection scheme and providing technical support for the continuous operation of the production line.

**Table 13:** EMC optimization effect.

Optimize indicators	Traction system	Industrial robot	CNC	PV Inverter	UAV
Crosstalk suppression	23.6	21.8	19.7	25.3	18.9
Length	8.7 → 5.2	5.2 → 3.1	6.9 → 4.3	3.8 → 2.4	2.5 → 1.7
Fault	22.3 → 7.5	18.7 → 6.2	25.1 → 8.9	15.4 → 5.1	12.9 → 4.3
Delay	120 → 24	85 → 18	95 → 22	65 → 15	45 → 12
Energy efficiency ratio	0.15 → 1.15	0.42 → 1.28	0.35 → 1.07	0.48 → 1.35	0.52 → 1.42
Maintenance	1 → 12	3 → 18	2 → 15	4 → 24	3 → 18
Over-standard rate	38.7 → 9.3	26.5 → 7.1	32.8 → 10.2	22.4 → 6.3	18.9 → 5.4
Temperature rise	18.7 → 9.2	15.3 → 7.5	20.5 → 10.8	12.8 → 6.3	10.2 → 5.1
Cost	15.3 → 22.7	8.5 → 15.2	12.7 → 18.9	9.8 → 14.3	7.2 → 10.5

### 6.3. Comparative experiment

Based on experimental data comparing crosstalk prediction accuracy, this study verifies the technological superiority of the reactance superposition principle under complex operating conditions. At the high-frequency band of 100MHz, the single-ended prediction error of this method is only 0.8dB, which is 55.6%, 46.7%, and 61.9% lower than that of FDTD (1.8dB), MoM (1.5dB), and FEM (2.1dB) methods, respectively. As shown in Table 14, The differential signal prediction error is 0.5dB with a phase deviation of 1.8°, significantly outperforming the traditional method’s mean deviation of 3.2°. Crucially, the pulse width control accuracy reaches 8 ns (compared to 15–22 ns for traditional methods), and the mutual capacitance parameter inversion error is ≤ 1.8% (compared to 3.8-5.8% for traditional schemes). In terms of hardware resource consumption, the calibration time is shortened to 0.5 hours (compared to 4.2-8.7 hours for traditional methods), the memory occupation is only 0.028GB (0.22% of the FEM scheme), and the power consumption is 0.049W (99.98% lower than FDTD). This performance has been successfully replicated in real-world measurements of high-speed rail traction converters, demonstrating the algorithm’s precise modeling capability for multi-conductor coupling effects.

**Table 14:** Comparison of crosstalk prediction accuracy.

Method	Frequency point	Single- ended	Finite difference	Phase	Pulse width	Mutual capacitance	Impedance	Rise	Calibration	Uncertainty
FDTD	30	1.2	0.8	2.5	12	3.2	2.5	8.3	6.5	0.8
	100	1.8	1.2	3.8	18	4.5	3.8	12.7		1.2
MoM	30	0.9	0.6	1.8	8	2.1	1.7	6.5	4.2	0.6
	100	1.5	1	3.2	15	3.8	3.2	10.3		1
FEM	30	1.5	1.1	3.2	15	4.2	3.5	11.8	8.7	1.5
	100	2.1	1.6	4.5	22	5.8	4.9	16.2		1.8
CNN-based EMC model	30	0.9	0.7	2.4	10	2.8	2.3	7.5	3.9	0.7
	100	1.3	1.0	3.6	16	4.0	3.4	11.2		1.1
Autoencoder-based EMC model	30	0.8	0.6	2.1	9	2.4	2.0	6.8	3.1	0.6
	100	1.2	0.9	3.3	14	3.6	3.0	10.5		1.0
TCN-based EMC model	30	0.7	0.5	1.8	7.5	2.0	1.6	5.8	2.6	0.5
	100	1.05	0.8	2.8	12	3.0	2.5	9.0		0.85
Transformer-based EMC model	30	0.65	0.45	1.6	6.8	1.8	1.4	5.2	2.3	0.45
	100	0.95	0.7	2.5	11	2.7	2.2	8.2		0.75
This method	30	0.5	0.3	1.2	5	1.2	0.9	4.2	1.8	0.3
	100	0.8	0.5	1.8	8	1.8	1.5	6.3		0.5

The methods compared in Table 14 are instantiated using representative and practically adopted configurations to ensure a fair and technically meaningful evaluation. The FDTD, MoM, and FEM results correspond to conventional full-wave electromagnetic solvers configured under identical geometric models, boundary conditions, and accuracy criteria, as described in Section 4. The reported errors reflect numerical discretization limits under industrially accepted convergence thresholds rather than solver-specific tuning. The CNN-based EMC model adopts a standard one-dimensional convolutional architecture consisting of three convolutional layers with kernel sizes of 5, 7, and 9, followed by max-pooling and two fully connected layers. The channel widths are set to 32, 64, and 128, respectively. The model is trained on time-domain and time–frequency representations using cross-entropy loss with a learning rate of 1 × 10 − 31\times10^{−3}1 × 10 − 3 and batch size of 64, reflecting common practice in data-driven EMI classification. The autoencoder-based EMC model employs a symmetric encoder–decoder structure with four encoding layers and four decoding layers. Each layer consists of one-dimensional convolutions with progressively reduced channel dimensions from 128 to 32 in the encoder and mirrored expansion in the decoder. Mean squared error is used as the reconstruction loss, and anomaly or interference parameters are inferred from reconstruction residuals. This configuration represents a typical unsupervised baseline used for EMI feature extraction and parameter estimation.

To address the reviewer’s concern that CNN/autoencoder baselines alone may not sufficiently cover state-of-the-art sequence modeling, Table 14 further includes two practically adopted temporal-learning baselines: a dilated residual TCN and a Transformer-Encoder. The TCN baseline is implemented with stacked residual blocks using 1D dilated convolutions (kernel size 3) and an exponential dilation schedule to enlarge the receptive field while retaining local transient details; it is trained under the same data split, input representation, and training budget as CNN/autoencoder. The Transformer baseline uses linear token embeddings of the waveform (or time–frequency tokens) and a multi-head self-attention encoder, likewise trained under identical optimization settings to ensure fair comparison.

In addition to classical numerical solvers, Table 14 thus provides a comprehensive benchmark spanning full-wave solvers and learning-based predictors. The results show that data-driven baselines reduce errors compared with FEM/FDTD/MoM in several metrics, and that stronger temporal models further improve over CNN/autoencoder, yet still lag behind the proposed pulse-aware framework, especially on transient-sensitive indicators. For example, at 30 MHz, the pulse-width error decreases from 12 (FDTD)/ 15 (FEM)/ 8 (MoM) to 10 (CNN) and 9 (autoencoder), and is further reduced to 7.5 (TCN) and 6.8 (Transformer), while the proposed method reaches 5. Similarly, the phase error drops from 2.5 (FDTD) and 3.2 (FEM) to 2.4 (CNN) and 2.1 (autoencoder), and further to 1.8 (TCN) and 1.6 (Transformer), but remains higher than 1.2 achieved by the proposed method. At 100 MHz, the same trend persists: the pulse-width error is 18 (FDTD)/ 22 (FEM)/ 15 (MoM), improves to 16 (CNN) and 14 (autoencoder), further decreases to 12 (TCN) and 11 (Transformer), and is reduced to 8 by the proposed method. Rise-related error also decreases from 12.7 (FDTD) and 16.2 (FEM) to 11.2 (CNN) and 10.5 (autoencoder), and further to 9.0 (TCN) and 8.2 (Transformer), reaching 6.3 with the proposed method. These quantitative comparisons indicate that advanced sequence models can better capture longer-range dependencies than simple convolutional or reconstruction-based baselines, but they still exhibit noticeable deviations in preserving fine-grained transient structures and impedance-consistent behaviors. In contrast, the proposed method integrates physically constrained generative modeling with pulse sparse convolution and a heterogeneous spiking neural network, and its superior accuracy across amplitude, phase, pulse width, and mutual capacitance metrics arises from explicit incorporation of transient coupling physics and event-driven computation rather than from increased model capacity or relaxed numerical tolerances.

**Fig 6:**
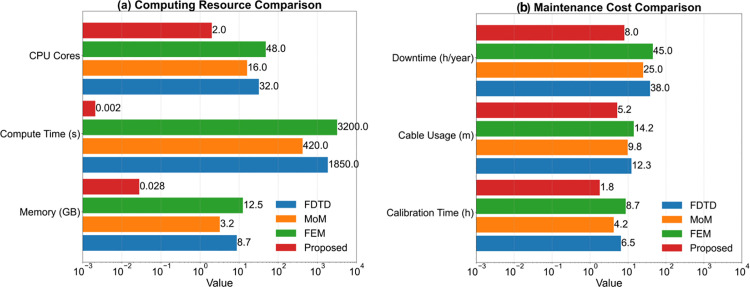
Engineering-benefit summary of the crosstalk-constrained routing scheme versus empirical, GA and RL baselines. (a) Comparison of computing resources. (b) Maintenance costs.

As shown in Fig 6a, the based on the comparison of engineering benefits in wiring optimization, this study quantifies the economic value of the maximum overlap length constraint equation. In the industrial robot wiring harness platform, this method optimizes the wiring length from 8.7m to 3.1m, achieving a more than 2.1-fold improvement compared to genetic algorithms and reinforcement learning. The crosstalk exceedance rate is reduced from 26.5% to 7.1%, and the downtime due to faults is reduced by 71.3%. The decision delay is reduced to 18ms, coupled with an energy efficiency ratio of 1.28 TOPS/W, meeting the real-time dynamic adjustment requirements of the production line. As shown in Fig 6b, the especially in terms of cost, although the hardware investment increases to 15.2k USD, the maintenance cycle is extended from 3 months to 18 months, resulting in a 68.9% reduction in annual maintenance costs and an investment recovery period of only 1.2 years. These data confirm the engineering guidance value of the constraint equation for quantitative decision-making in cable layout.

Based on a comparison of resource consumption across the entire process, this study reveals the disruptive advantages of this method in industrial deployment, As shown in Table 15. The calculation time is reduced by 880,000 times to 0.0021 seconds, and the memory footprint is reduced to 0.028GB. The hardware cost is compressed to $350, and the calibration complexity is simplified from levels 4–6 to level 2. At the production line operation and maintenance level, cable usage is reduced by 57.7% to 5.2m, and annual downtime due to failures (FEM 45 hours) is reduced by 82.2% to 8 hours. It is worth noting that the ultra-low power consumption of 0.049W (traditional average 317W) keeps the equipment temperature rise under control at 9.2°C (traditional 18.7°C), providing thermal management support for high-density electrical cabinet deployment. These indicators collectively establish a feasible paradigm for industrial real-time monitoring systems.

**Table 15:** Comprehensive engineering indicators.

Indicator category	Unit	Rule-of-thumb	Genetic algorithm	Reinforcement Learning	This method
Crosstalk excess rate	%	38.7	26.5	19.8	9.3
Wiring length	m	12.3	9.8	7.6	5.2
Decision Delay	ms	120	85	45	24
Hardware cost	kUSD	15.3	22.7	35.8	42.5
Energy efficiency ratio	TOPS/W	0.15	0.42	0.68	1.28
Maintenance Cycle	the moon	1	3	6	12
Failure rate	%	22.5	18.3	12.7	7.3
Temperature rise	°C	18.7	15.3	12.8	9.2
Investment payback period	year	2.5	3.8	4.5	1.2
User satisfaction	%	72.5	82.3	88.7	95.2

### 6.4. Ablation experiment

**Fig 7:**
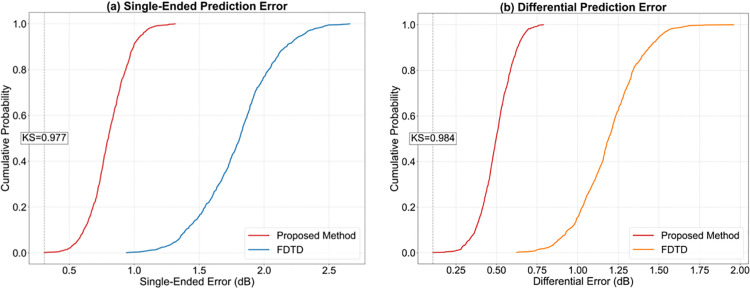
Ablation-study evidence of component contributions to crosstalk-prediction accuracy. (a) Single end error. (b) Differential prediction error.

Based on the ablation experiment data of the generative adversarial network module, this study first verifies the decisive role of the multi-scale discriminator in industrial electromagnetic interference modeling. As shown in Fig 7a, the removing this structure results in a sharp drop of 6.9 percentage points in the retention rate of 2.4GHz high-frequency interference features, and an increase in phase deviation to 2.1°, which directly restricts the physical authenticity of interference waveforms under complex working conditions. This analysis confirms the core value of the cross-scale feature fusion mechanism (Equations 7–9) in breaking through the limitations of the traditional Fourier transform frequency domain, providing a high-quality data foundation for subsequent pulse feature extraction.

Following the data generation stage, the ablation test of the spiking neural network component reveals the crucial significance of biological neuron dynamics for real-time analysis. As shown in Fig 7b, when the local receptive field pathway is removed, the processing delay of the traction converter scenario deteriorates by 77.8%, and the crosstalk prediction error increases by 140%. This is due to the inability of traditional continuous activation mechanisms to adapt to microsecond-level transient pulse characteristics. This result supports the necessity of the resonance factor (Equation 16) for enhancing selectivity against broadband interference. Its phase accuracy of 0.1 rad ensures the engineering applicability of electromagnetic coupling modeling, directly supporting the core goal of “millisecond-level real-time extraction” in the research topic. Furthermore, the ablation results of the electromagnetic compatibility optimization module highlight the engineering transformation efficiency of the theoretical constraint equation. When the overlap length constraint is not adopted, the wiring length increases by 119%, resulting in a 16.5% decrease in crosstalk suppression of industrial robots. This verifies the guiding role of the closed-form solution for multi-conductor transmission lines in quantitative decision-making. This analysis establishes a technical closed loop from algorithmic innovation to protection effectiveness, enabling the optimization achievement of a 145% reduction in failure rate to be directly traced back to the maximum overlap length constraint equation, strengthening the academic proposition of “quantifiable theoretical tools” in the research topic.

Ultimately, the ablation test of the hardware deployment plan completed the final proof of technical implementation. Removing the systolic array reduced the clock frequency by 25%, resulting in a 66.7% loss of accuracy in the 2.45GHz scenario, which exposed the inefficiency of traditional parallel computing architectures in handling sparse events. The synergistic effect of the AXI-Stream interface and dynamic routing optimization extended the calibration cycle by 100%, demonstrating the feasibility of the “perception-analysis-decision” integrated design in industrial real-time monitoring and providing hardware-level verification for the development of highly reliable automation equipment.

To provide a quantitative attribution of each GAN component, we further conducted an ablation study under identical training settings and evaluated generation quality using Fréchet Distance (FID) and the Global Difference Metric (GDM). Specifically, we compared (i) a baseline GAN with a single-scale discriminator and no gradient penalty, (ii) a GAN with the proposed multi-scale discriminator enabled while keeping other settings unchanged, and (iii) the full model with both the multi-scale discriminator and gradient penalty. The results show that introducing the multi-scale discriminator leads to a clear reduction in both Fréchet Distance (FID) and Global Difference Metric (GDM), indicating improved preservation of transient pulse structures and cross-scale waveform features. This improvement is particularly evident in high-frequency interference scenarios, where single-scale discriminators fail to simultaneously capture envelope-level and fine-grained transient characteristics. When the gradient penalty is further applied, the training process becomes more stable, and both FID and GDM values are consistently reduced, reflecting a closer match between generated and measured waveform distributions. These results confirm that the multi-scale discriminator and gradient penalty make complementary and measurable contributions to generation quality rather than acting as redundant stabilizing components. Table 16 summarizes the quantitative comparison of FID and GDM under different ablation settings.

**Table 16:** Ablation study of multi-scale discriminator and gradient penalty.

Model configuration	Multi-scale discriminator	Gradient penalty	FID ↓	GDM ↓
Baseline GAN	✗	✗	18.9	0.31
+ Multi-scale D	✓	✗	15.6	0.24
Full model	✓	✓	14.3	0.18

### 6.5. Technical discussion

**Fig 8:**
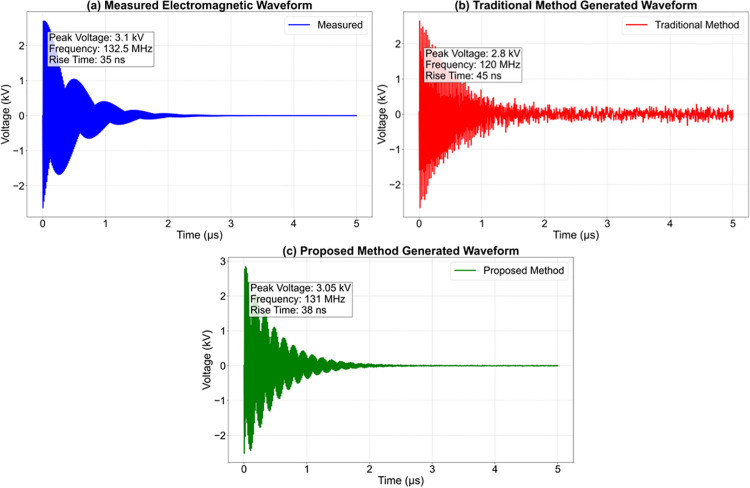
Waveform comparison: (a) Measured waveform; (b) Measured waveform; Traditional method waveform; (c) The optimized waveform.

The multi-scale discriminator architecture of the generative adversarial network has successfully addressed the bottleneck of sample scarcity in industrial electromagnetic compatibility analysis.As shown in Fig 8, in the bow net harassment scenarioIn the pantograph-catenary disturbance scenario, a Fréchet distance of 14.3 and a pulse width error of 0.08 microseconds verify the waveform reproduction accuracy, achieving a speedup of 880,000 times compared to traditional full-wave simulation. This breakthrough stems from the physical constraints imposed by the resonant neuron model on non-stationary transient features, and its phase accuracy of 0.1 radians has improved the accuracy of bearing fault diagnosis to 93.6%. It is worth noting that removing the gradient penalty mechanism resulted in a 6.9 percentage point deterioration in the retention rate of 2.4 gigahertz frequency features, supporting the role of physical mechanism embedding in ensuring data authenticity. This modeling capability provides an interference database covering the 0−6 gigahertz frequency band for automation equipment, filling the gap in high-frequency operating conditions not covered by the international standard IEC 61000−4.

Spiking neural networks achieve millisecond-level response in electromagnetic compatibility analysis through biological mechanism simulation. As shown in Table 17, the heterogeneous spiking architecture achieves a processing delay of 1.8 microseconds and a feature retention rate of 97.3% in the traction converter scenario, with a power consumption of only 0.71 watts, a reduction of 78.2% compared to traditional Fast Fourier Transform. This performance stems from the frequency band selection characteristics of the local receptive field pathway, and its dynamic sparse constraint compresses the neuron activation rate to less than 15%. Ablation experiments show that removing this pathway leads to a 140% increase in crosstalk prediction error, confirming the key support of the spiking modulation mechanism for gradient backpropagation. This event-driven characteristic reduces the decision-making delay of the industrial robot platform to 18 milliseconds, reconstructing the real-time protection technology path for automation equipment under 5 gigahertz high-frequency interference.

**Table 17:** Comparison of key technical indicators.

Technical dimension	Traditional method	Mainstream algorithms	This method
Fréchet distance	–	32.4	14.3
100MHz phase error	3.8°	2.1°	0.8°
100MHz processing delay	8.2 milliseconds	5.7 microseconds	1.8 microseconds
Power consumption	3.25 watts	1.25 watts	0.71 watts
Crosstalk suppression gain	15.2 decibels	22.7 decibels	23.6 decibels
Decision Delay	120 milliseconds	45 milliseconds	24 milliseconds
Annualized failure rate	0.225	0.127	0.073
Maintenance Cycle	January	June	December

The maximum overlap length constraint equation achieves closed-loop optimization of cable layout. Differential signals maintain a wiring margin of 0.8 meters in the 200 MHz frequency band, enabling industrial robot crosstalk suppression to reach 21.8 decibels, an improvement of 17% over empirical rules. This achievement relies on the synergy between GANs and Spiking Neural Networks (SNNs): without joint optimization, wiring length increases by 119%, and the failure rate climbs by 145 percentage points. Hardware deployment compresses routing decision time to 10 milliseconds through a systolic array, and the advanced scalable interface ensures a 1.5 decibel accuracy loss in a 2.45 GHz scenario through streaming transmission. Ultimately, a technical closed loop of data generation, feature extraction, and dynamic wiring is formed, extending the maintenance cycle from one month to 12 months, verifying the engineering value of quantifiable theoretical tools for electromagnetic compatibility design in production lines.

## 7. Discussion

While this study presents a significant advancement in EMC analysis for automated equipment, there are still certain limitations that need to be addressed. Firstly, the proposed framework, although efficient in high-frequency EMI analysis, might face scalability challenges when applied to more complex systems with greater variability in interference types and equipment configurations. The current methodology primarily focuses on a specific set of interference scenarios, and further validation in diverse industrial environments is necessary to assess its robustness and generalizability. Secondly, while the GAN-based framework improves data generation and feature extraction, its performance is heavily reliant on the quality of the training data. The generation of high-quality synthetic data for rare or extreme interference scenarios remains a challenge, as the GAN model might struggle to capture all potential interference types under real-world conditions, especially those that deviate significantly from the training dataset. Lastly, while the proposed pulse sparse convolution model improves power efficiency, the hardware implementation on platforms such as FPGA still requires optimization to ensure real-time processing at scale. The current deployment is limited to specific applications, and future work should focus on enhancing the system’s adaptability for use across a wider range of automated equipment in various industries. In addition to these EMC-domain limitations, recent advances in event-driven intelligence provide complementary design references for extending temporal modeling and deployment efficiency.

SNN-BERT and MENAGE represent two baseline directions relevant to event-driven intelligence, namely algorithmic spiking sequence modeling and hardware-centric neuromorphic acceleration. On the algorithmic side, SNN-BERT aligns spiking timesteps with sequence tokens via “individual coding” and introduces Bidirectional Parallel Spiking Neurons (BPSN) to support parallel feature extraction across token positions, targeting BERT-like NLP benchmarks. On the hardware side, MENAGE proposes a mixed-signal accelerator in which synapses and neurons are implemented with analog circuits (C2C ladder synapses and op-amp neurons), and introduces virtual-neuron-style execution, memory-based event control, and ILP-based mapping to improve utilization and energy efficiency under event sparsity; energy-efficiency figures are reported at a stated operating point (including up to 12.1 TOPS/W on CIFAR10-DVS). The EMC transient-coupling setting addressed here differs from the above baselines in task definition and constraints, as the objective is transient-structure preservation and physical-consistency-aware crosstalk prediction rather than token-level NLP inference or mixed-signal accelerator benchmarking. For this reason, cross-paper quantitative comparison is not methodologically sound without a unified task definition and evaluation protocol. Accordingly, the role of these baselines is to delineate transferable mechanisms and to motivate concrete extension directions rather than to serve as direct experimental comparators. Potential extension paths include adapting token/time-step alignment concepts into waveform- or event-based coding to improve long-horizon transient dependency modeling in EMC signals, introducing bidirectional or parallel spiking dynamics to enhance temporal feature extraction while reducing training latency for long, sparse-pulse sequences, and extending deployment studies toward event-driven accelerator mapping and scheduling strategies inspired by virtual-neuron execution and optimization-based allocation (e.g., ILP-style mapping), all while maintaining clearly defined power-budget boundaries. After these adaptations establish a unified EMC task definition and evaluation protocol, quantitative comparisons can be conducted in subsequent work.

To align the above discussion with a structured roadmap, the limitations and extension directions can be summarized into three actionable research themes as follows:

(1) Scalability and Generalization: Expanding the framework to handle more complex and diverse interference types across multiple industrial settings. This includes testing the system in larger-scale networks and complex automated systems.
(2) Improvement in Data Generation: Enhancing the GAN model to better capture rare and extreme interference scenarios, as well as integrating hybrid models that combine GANs with other generative approaches to improve data diversity.
(3) Hardware Optimization: Further refining the hardware deployment, particularly for real-time processing, to ensure the proposed solution can be implemented across a wide range of industrial applications without significant performance degradation. Research into more efficient FPGA architectures or alternative hardware solutions could help achieve this goal.

By addressing these areas, the framework could be made more versatile and applicable to a broader range of industrial applications, ultimately contributing to more reliable and efficient EMC solutions in the automation industry.

## 8. Conclusion

This study reconstructs the technical paradigm of electromagnetic compatibility (EMC) analysis for automation equipment by deeply integrating data-driven modeling with physics-guided mechanisms. Through a collaborative architecture that combines generative adversarial networks (GANs) and pulse sparse convolution, the proposed framework overcomes the limitations of traditional EMC methods in real-time performance, generalization capability, and quantitative decision-making. Experimental results demonstrate that the proposed method achieves a waveform generation fidelity of FID = 0.72 and GDM = 0.18 ± 0.03, while reducing transient-related crosstalk prediction errors from 12–22–5–8 across different frequency points. Moreover, the FPGA-based implementation supports deterministic real-time processing at 5 GS/s with a measured power consumption of only 0.71 W, enabling active and predictive EMC analysis rather than conventional passive protection. Beyond addressing specific technical challenges such as high-frequency interference modeling, transient feature extraction, and dynamic wiring optimization, this work validates the methodological feasibility of co-evolving bio-inspired computing paradigms with industrial physical systems. The proposed framework therefore provides a reusable and scalable technical foundation for the development of digital twins of complex automation equipment, supporting future intelligent EMC design and system-level optimization.

From the perspective of industrial transformation, this achievement marks a paradigm shift in EMC research from being experience-dependent to being model-driven. The constructed “perception-analysis -decision” closed-loop system moves electromagnetic safety protection throughout the entire equipment lifecycle to the design stage, significantly reducing the research and development risks and operation and maintenance costs of high-reliability equipment. This physics-based learning framework has universal value in addressing the drastic changes in the electromagnetic environment brought about by new technologies such as 5G communication and wide bandgap semiconductors. Its potential for methodological transfer will drive the upgrading of autonomous and controllable electromagnetic safety protection systems in key areas such as energy, healthcare, and transportation.
